# Hepatitis B Virus Genotype E/A Recombinants from Blood Donors in Beira, Mozambique

**DOI:** 10.1128/mra.00182-23

**Published:** 2023-05-25

**Authors:** Barbara Tyler, Heidi L. Meeds, Elizabeth A. Odegard, Nalia Ismael, Adolfo Vubil, Ana Flora Zicai, Nédio Mabunda, Jason T. Blackard

**Affiliations:** a Division of Digestive Diseases, University of Cincinnati College of Medicine, Cincinnati, Ohio, USA; b Instituto Nacional de Saúde, Marracuene, Mozambique; DOE Joint Genome Institute

## Abstract

Hepatitis B virus (HBV) is endemic in many parts of sub-Saharan Africa. Here, we present 5 full-length HBV recombinant genomes from blood donors in Beira, Mozambique. The genomes are recombinants between genotypes E and A and are distantly related to one another, based on their genetic distances.

## ANNOUNCEMENT

Hepatitis B virus (HBV) (family *Hepadnaviridae*, genus *Orthohepadnavirus*) is a viral infection of the liver that can lead to cirrhosis and hepatocellular carcinoma. HBV infection is endemic in many sub-Saharan African countries. The virus contains a relaxed circular, partially double-stranded DNA genome that is ~3,200 bases in length. The genome includes four open reading frames (ORFs), coding for the polymerase (P), surface (S), core (C), and X proteins (1). HBV can be classified into 10 genotypes (genotypes A through J), and intergenotypic recombination has been reported (2–7). As in other sub-Saharan African countries, HBV is endemic in Mozambique (8, 9); however, few studies have examined HBV genotypes or recombination in Mozambique. Here, we present 5 full-length HBV genotype E/A recombinant viruses from blood donors in Beira, Mozambique.

In a previous study approved by the National Bioethics Committee in Mozambique (approval number 263/CNBS/2014), we evaluated partial HBV genomes from 57 HBV DNA-positive blood donor samples that were collected between November 2015 and April 2016. The majority of the viruses were nonrecombinant genotype A viruses, while 6 were putative recombinant viruses (10). In the current study, full-length genomes of these putative recombinant viruses were amplified and analyzed further. Viral DNA was extracted from blood donor plasma using the QIAmp UltraSens virus kit. The extracted DNA underwent a completion/ligation (C/L) step using the C/L reagents CutSmart buffer, bovine serum albumin (BSA), deoxynucleoside triphosphates (dNTPs), ATP, T4 DNA polymerase, and T4 DNA ligase, followed by a rolling circle amplification (RCA) step using the TempliPhi kit from Cytiva Life Sciences. Full-length HBV PCR was conducted using PicoMaxx high-fidelity PCR master mix (Agilent), HBV forward primer 5′-CCG GAA AGC TTG AGC TCT TCT TTT TCA CCT CTG CCT AAT CA-3′, and HBV reverse primer 5′-CCG GAA AGC TTG AGC TCT TCA AAA AGT TGC ATG GTG CTG G-3′. Amplified PCR products were run on a 1% agarose gel, and 3.2-kb products were cut and extracted using the QIAEX II gel extraction kit.

PCR products were submitted to the University of Cincinnati College of Medicine Genomics, Epigenomics, and Sequencing Core for next-generation sequencing (NGS). The New England BioLabs NEBNext Ultra II FS DNA library preparation kit was used to prepare the library, and sequencing was performed on an Illumina HiSeq 1000 sequencer with 1 × 51-bp single reads. A consensus sequence was generated by mapping the raw reads to the reference genome (GenBank accession number AY233282) from South Africa using UGENE v44.0 (11). Of note, a small portion of the full-length genome sequence corresponds to the primers utilized to amplify the virus and was not determined *de novo*. Consensus sequences were aligned to reference genomes from the Hepatitis Virus Diversity Research (HVDR) database using Clustal X v2.1 (12, 13). The alignment was visualized in FigTree v1.4.4. Consensus sequences were evaluated for recombination using the jumping profile Hidden Markov Model (jpHMM) program (14). Genetic distances were calculated in MEGA11 with the Kimura two-parameter model (15). All tools were run with default parameters unless otherwise specified. Details about raw reads and consensus sequences are available in Table 1.

**TABLE 1:** HBV recombinant genome characteristics

Patient identification no.	GenBank accession no.	No. of raw reads	Avg read depth (×)	Consensus length (bp)	Consensus GC content (%)
BSB0632	OP514931	376,456	6,495	3,002	49.1
BSB0670	OP514932	599,795	9,635	3,175	48.4
BSB1014	OP514933	318,498	5,079	3,198	48.3
BSB1230	OP514934	388,153	6,161	3,213	48.4
BSB1364	OP514935	226,661	3,762	3,073	48.2

**FIG 1:**
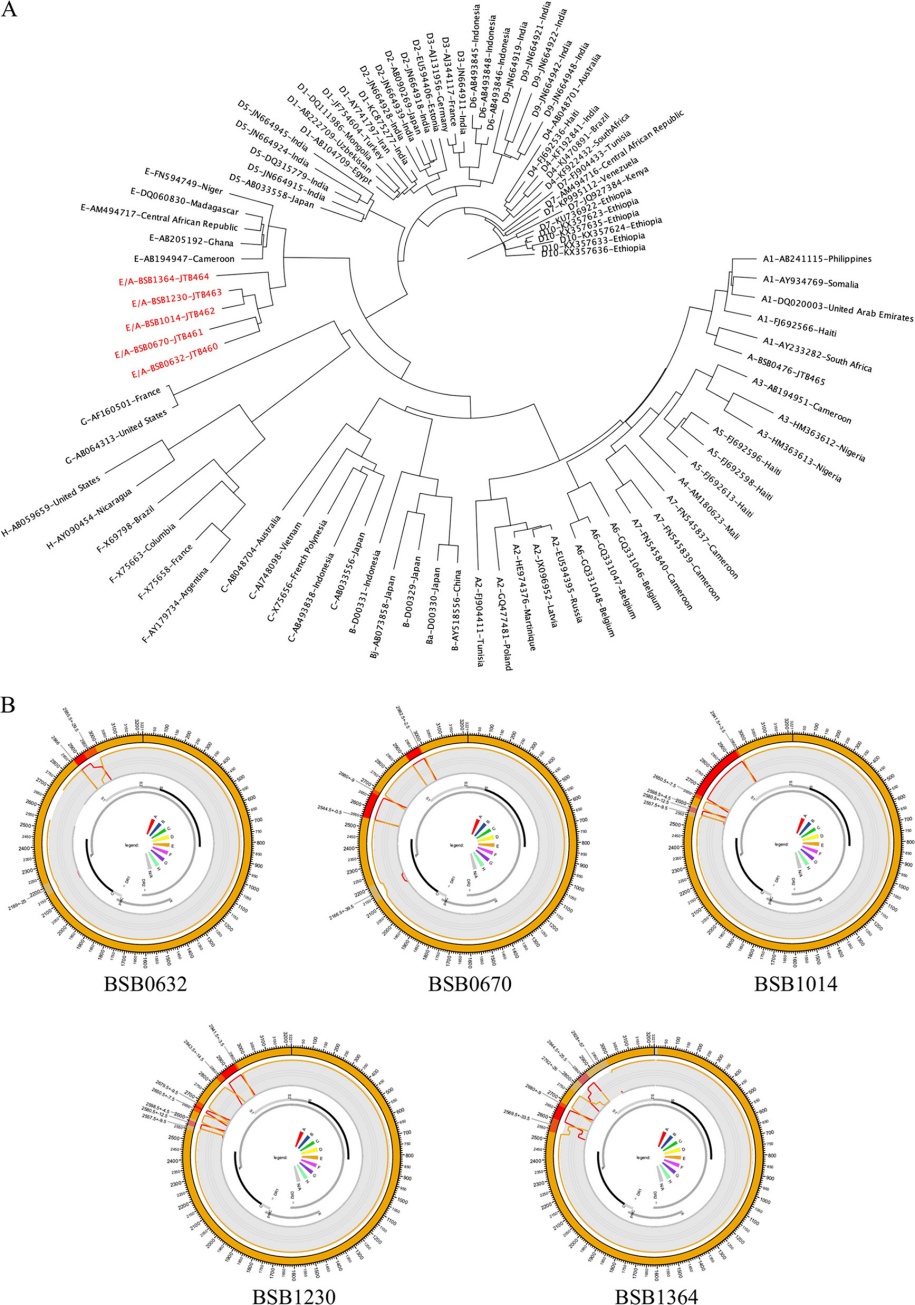
(A) Phylogenetic tree of full-length recombinant sequences from Mozambique. Each sequence is labeled with its GenBank accession number, country of origin, and genotype. Full-length recombinant sequences from Mozambique are colored in red. (B) Recombination prediction profiles. Profiles were evaluated using the jpHMM program, which employs a probabilistic approach to compare a nucleic acid or protein sequence S to a given multiple alignment A of a sequence family for which a classification into subtypes is available. jpHMM does not align and compare a database sequence S to the multiple alignment A as a whole; rather, it aligns local segments of S to those segments of individual sequences from A that are most similar to them. Recombination analysis of circular genomes is performed with artificially linearized sequences of the circular genomes using linear models. Samples are identified by their patient identification number.

Of the 6 samples that were previously identified as putative recombinants, 1 sample could not be amplified by full-length PCR. The 5 putative recombinant viruses from Mozambique clustered separately from all genotype E references, as shown in Fig. 1A. The recombination prediction profiles confirmed that these samples were genotype E/A recombinants, with much of their genomes belonging to genotype E (Fig. 1B). The average genetic distance for these 5 samples was 2.58% (range, 1.17 to 3.49%), compared to 1.83% (range, 0.23 to 3.43%) for 14 nonrecombinant genotype E samples from Ghana (16, 17). Recombinant E/A isolates have been reported previously in West and Central African countries (4–7, 18–22), although the current study highlights the broader range of these unique variants.

### Data availability.

The raw sequence data are available under BioProject PRJNA885128, with SRA accession numbers SRR21743134 through SRR21743138. The consensus HBV genomes are available in GenBank under accession numbers OP514931 through OP514935.
